# ECMO support as a bridge to lung transplantation is an independent risk factor for bronchial anastomotic dehiscence

**DOI:** 10.1186/s12890-022-02280-9

**Published:** 2022-12-20

**Authors:** Enora Atchade, Mélissa Ren, Sylvain Jean-Baptiste, Alexy Tran Dinh, Sébastien Tanaka, Parvine Tashk, Brice Lortat-Jacob, Maksud Assadi, Gaelle Weisenburger, Hervé Mal, Jean Nicolas Sénémaud, Yves Castier, Christian de Tymowski, Philippe Montravers

**Affiliations:** 1 APHP, CHU Bichat-Claude Bernard, DMU PARABOL, 46 Rue Henri Huchard, 75018 Paris, France; 2 INSERM U1148, LVTS, CHU Bichat-Claude Bernard, 46 Rue Henri Huchard, 75018 Paris, France; 3 Université de Paris, UFR Diderot, Paris, France; 4 INSERM UMR 1188, Diabète Athérothrombose Réunion Océan Indien (DéTROI), Université de La Réunion, Saint-Denis de La Réunion, France; 5 Service de Pneumologie B et Transplantation Pulmonaire, APHP, CHU Bichat-Claude Bernard, 46 Rue Henri Huchard, 75018 Paris, France; 6 Service de Chirurgie Thoracique et Vasculaire, APHP, CHU Bichat-Claude Bernard, 46 Rue Henri Huchard, 75018 Paris, France; 7 INSERM UMR 1149, Immunorecepteur et Immunopathologie Rénale, CHU Bichat-Claude Bernard, 46 Rue Henri Huchard, 75018 Paris, France; 8 Physiopathologie et Epidémiologie des Maladies Respiratoires, INSERM UMR 1152, Paris, France

**Keywords:** Lung transplantation, Bronchial anastomotic dehiscence, Airway complications, Extra corporeal membrane oxygenation

## Abstract

**Background:**

Airway complications are frequent after lung transplantation (LT), as they affect up to 23% of recipients. The implication of perioperative extracorporeal membrane oxygenation (ECMO) support and haemodynamic instability has never been specifically assessed. The first aim of this study was to explore the impact of perioperative ECMO support on bronchial anastomotic dehiscence (BAD) at Day 90 after LT.

**Methods:**

This prospective observational monocentric study analysed BAD in all consecutive patients who underwent LT in the Bichat Claude Bernard Hospital, Paris, France, between January 2016 and May 2019. BAD visible on bronchial endoscopy and/or tomodensitometry was recorded. A univariate analysis was performed (Fisher’s exacts and Mann–Whitney tests), followed by a multivariate analysis to assess independent risk factors for BAD during the first 90 days after LT (*p* < 0.05 as significant). The Paris North Hospitals Institutional Review Board approved the study.

**Results:**

A total of 156 patients were analysed. BAD was observed in the first 90 days in 42 (27%) patients and was the main cause of death in 22 (14%) patients. BAD occurred during the first month after surgery in 34/42 (81%) patients. ECMO support was used as a bridge to LT, during and after surgery in 9 (6%), 117 (75%) and 40 (27%) patients, respectively. On multivariate analysis, ECMO as a bridge to LT (*p* = 0.04) and septic shock (*p* = 0.01) were independent risk factors for BAD.

**Conclusion:**

ECMO as a bridge to LT is an independent risk factor for BAD during the first 90 days after surgery. Close monitoring of bronchial conditions must be performed in these high-risk recipients.

**Supplementary Information:**

The online version contains supplementary material available at 10.1186/s12890-022-02280-9.

## Introduction

Airway complications are frequent after lung transplantation (LT), affecting up to 23% of recipients [1–7]. Prior studies reported a related threefold increase in mortality after LT and a 30% decrease in the cumulative risk of survival [4]. The incidence of bronchial anastomotic dehiscence (BAD) has been estimated to be between 1 and 10% [2, 7, 8], with an attributable mortality between 2 and 5% [6, 9–11].

Risk factors for airway complications after LT have been assessed by prior retrospective studies, which evidenced risk factors linked to the donor (size mismatch between graft and recipient [1, 12], prolonged mechanical ventilation (MV) > 50 to 70 h [12], right-side transplantation [13], to the recipient (age > 54, primary graft dysfunction (PGD), acute rejection during the first month [5], microbial infection during the first trimester, preoperative colonization of the recipient, PaO_2_/FiO_2_ ratio < 300 during the first 72 h [14], presence of *Aspergillus fumigatus* in the respiratory tract during the first month, sirolimus as immunosuppressive treatment [15], and to the surgical technique (telescoped anastomoses being associated with an increased incidence of bronchial anastomotic complications compared with end-to-end anastomoses [1, 3, 9, 12, 16].

Anastomotic complications after LT are related to bronchial ischaemia [2] and are linked to the intraoperative section of bronchial arteries. In the early postoperative period, bronchial vascularization exclusively depends on retrograde flow from pulmonary arteries, bringing less oxygenated blood [17]. After 2–4 weeks, anastomosis between pulmonary veins and bronchial arteries appears [7, 17], but their growth stops most often at the level of the bronchial anastomosis [18]. Haemodynamic instability, defined as an imbalance between oxygen supply and requirement, could be implicated in BAD occurrence by worsening bronchial ischaemia. The perioperative period is at high risk for haemodynamic instability because of the preexisting condition of the recipients (severe hypoxemia, right ventricle dysfunction, pulmonary hypertension), which is worsened by intraoperative events (clamping of pulmonary arteries, major bleeding resulting in hypovolemia and vasoplegia, left atrium suture resulting in a decreased cardiac preload and cardiac output, and air embolism of the coronary arteries at pulmonary arterial declamping). Management of perioperative haemodynamic instability often relies on ECMO support (used in 42–56% of the cases during the LT procedure [19–21], and only 45% of the recipients are weaned at the end of the surgical intervention [21, 22] for a mean duration of 1.5–3 days [21, 22].

The first aim of this study was to assess the association between perioperative ECMO support and BAD occurrence during the first 90 days after LT. The secondary aims were to study the incidence of BAD and the outcome of recipients with BAD and to explore the impact of perioperative haemodynamic instability on BAD occurrence during the first 90 days after LT.

## Materials and methods

### Study population

This prospective, observational, monocentric study analysed all consecutive patients who underwent LT at Bichat Claude Bernard Hospital between January 2016 and May 2019. This study was conducted in accordance with the declaration of Helsinki. The Paris-North-Hospitals Institutional Review Board reviewed and approved the study (IRB00006477). According to French law, due to the observational design of this study, the ethics committee waived the need for an informed consent.

### Data collection

The BAD of LT recipients, visible on bronchial endoscopy and/or tomodensitometry during the first 90 days after LT, was prospectively recorded. Baseline characteristics of the recipients (demographic data, underlying disease and comorbidities, type of LT procedure), data linked to the haemodynamic status of the recipient (ECMO support and type before, during and after surgery, duration, right ventricular dysfunction before surgery, catecholamine administration, dosage and duration, vascular filling, transfusion, severity scores and lactatemia on ICU admission, shock or AKI during ICU hospitalization), data linked to the haemodynamic status of the donor (catecholamine administration and dosage, transfusion, cardiac arrest), and outcome data (duration of MV and hospitalization in ICU, death in ICU, at Day 90 or during the first year after LT) were prospectively collected. High-dosage catecholamine administration was defined as norepinephrine or epinephrine administration > 0.5 µg/kg/min.

### Perioperative management

Perioperative care was standardized for all recipients according to our local protocol [23, 24]. Before surgery, ECMO as a bridge to LT is implemented in cases of persistent severe hypoxemia, despite high-flow oxygen therapy administration. Veno-venous ECMO is favoured in the absence of severe arterial pulmonary hypertension (mean pulmonary arterial pressure (PAP) measured by transthoracic echocardiography > 50 mmHg). During the intraoperative period, haemodynamic status is monitored and optimized using invasive arterial blood pressure, central venous and Swan Ganz catheters, and transoesophageal echocardiography. A venoarterial ECMO is implemented in cases of severe pulmonary arterial hypertension (mean PAP > 50 mmHg, despite NO administration), SaO_2_ < 85%, SvO_2_ < 60%, cardiac output < 1.5 L/min/m^2^ when clamping the pulmonary artery, if the patient does not tolerate single-lung ventilation (hypoxemia or hypercapnia), or in case of respiratory failure after transplantation of the first lung. Veno-venous ECMO as a bridge to LT is most often converted into a venoarterial device. Vascular filling is adapted to cardiac output and cardiac index variation and SvO_2_ and lactate measurements. Catecholamines (norepinephrine in first intention) are administered with a mean arterial blood pressure objective > 65 mmHg. Transfusion is administered under a low transfusion threshold (Hb < 7 g/dL). An autotransfusion system (Cell-saver®, Fresenius, Bad Homburg vor der Höhe, Germany) is used in the absence of contraindication. Intraoperative ECMO is not weaned immediately after surgery in cases of PaO_2_/FiO_2_ ratio < 200, SvO_2_ < 60%, mean PAP > 30 mmHg, or if transoesophageal echocardiography shows a right ventricular dysfunction. In the early postoperative period, bronchial monitoring relies on bronchial endoscopies performed every 48 h and more often in cases of clinical or radiological suspicion of pneumonia.

### Surgical technique

The bronchial anastomosis technique is standardized. On the left side, a limited dissection of the recipient bronchus and a section close to the mediastinum were performed. On the right side, a limited dissection of the recipient bronchus and a section at one ring or less of the birth of the upper lobar bronchi were performed. Anastomosis was performed without intussusception and buried in the peribronchial lymph node tissue on the right side and under the posterior pericardium on the left side. Intraoperative endoscopic control of bronchial anastomosis was performed.

### Statistical analysis

Risk factors for BAD were first studied on univariate analysis. Qualitative data were expressed as absolute numbers and percentages and compared by *χ*2 or Fisher’s exact test. Quantitative data were expressed as medians and interquartile ranges (IQRs) and were compared by the Mann–Whitney test. A sensitivity analysis was performed by excluding the recipients who died before Day 90 from a cause other than BAD. A multivariate analysis was performed using a logistic regression model to identify independent risk factors for BAD. The 90-days survival of LT recipients according to BAD was studied by Kaplan–Meier curves and compared by log-rank tests. *p* < 0.05 was defined as significant.

## Results

### General characteristics of the study population

**Fig. 1:**
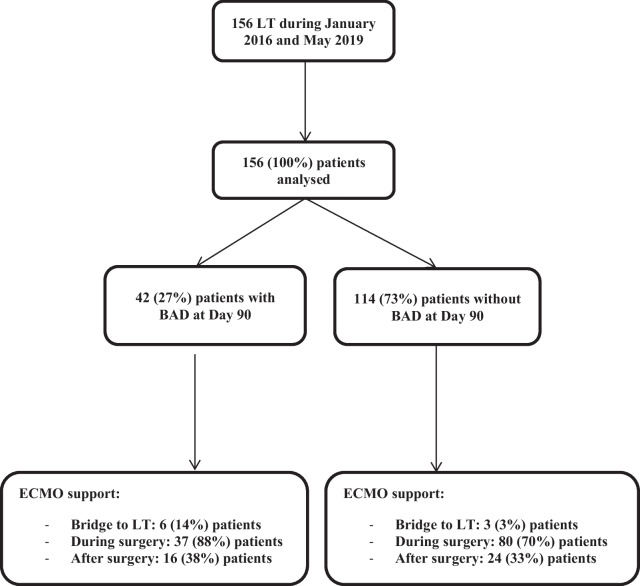
Flow chart of the study

Between January 2016 and May 2019, 156 patients underwent LT at Bichat Claude Bernard Hospital. All of them were included in the analysis. The flow chart of the study is presented in Fig. 1. The general characteristics of the study population are presented in Table 1.

**Table 1:** General characteristics of the study population

	All cohort n = 156	BAD at Day 90 n = 42 (27)	No BAD at Day 90 n = 114 (73)	p
Preoperative characteristics				
Male sex, n (%)	102 (65)	30 (71)	72 (63)	0.45
Age, median [IQR]	56 [50–62]	57 [51–60]	56 [50–62]	0.92
BMI, median [IQR]	24 [20–27]	24 [23–27]	23 [20–26]	0.08
Comorbidities, n (%)				
HBP	35 (22)	10 (24)	25 (22)	0.83
Diabetes mellitus	14 (9)	6 (14)	8 (7)	0.20
Dyslipidaemia	41 (26)	13 (31)	28 (25)	0.42
Ischaemic heart disease	11 (7)	1 (2)	10 (9)	0,29
Peripheral artery occlusive disease	10 (7)	3 (7)	7 (6)	1.00
Pulmonary hypertension	81 (54)	20 (50)	61 (55)	0.71
Right ventricle dilatation before LT	42 (27)	13 (31)	29 (25)	0.54
Underlying disease, n (%)				
COPD	55 (35)	13 (31)	42 (37)	0.57
PF	64 (41)	18 (43)	46 (41)	0.86
Other	37 (24)	11 (26)	26 (23)	0.68
Need for MV before surgery, n (%)	6 (4)	2 (5)	4 (3,5)	0.66
High-emergency LT, n (%)	26 (17)	10 (24)	16 (14)	0.15
Retransplantation, n (%)	4 (3)	3 (7)	1 (1)	0.06
Procedure				
Bilateral LT, n (%)	107 (67)	33 (79)	74 (65)	0.12
Postoperative variables				
SOFA score on ICU admission, median [IQR]	7 [5–8]	7 [5–8, 5]	6 [5–8]	0.36
SAPS II score on ICU admission, median [IQR]	36 [26–48]	38 [28–52]	36 [25–47]	0.15
Neuromuscular blocking agent administration, n (%)	46 (30)	20 (48)	26 (23)	0.01
Prone positioning, n (%)	18 (12)	7 (17)	11 (10)	0.26
PGD, n (%)	85 (55)	24 (57)	61 (54)	0.72
Number of pneumonia, median [IQR]	1 [1–2]	2 [1–3]	1 [1–1]	0.001
Acute cellular rejection, n (%)	21 (14)	7 (17)	14 (12)	0.60
Antibody mediated rejection, n (%)	44 (29)	18 (43)	26 (23)	0.03
Surgical thoracic reintervention, n (%)	22 (14)	10 (24)	12 (11)	0.04
Abdominal surgery, n (%)	16 (10)	8 (19)	8 (7)	0.04
Tracheostomy for ventilation weaning, n (%)	32 (21)	19 (45)	13 (12)	< 0.0001
Outcome				
Duration of MV, median [IQR]	3 [1–10, 5]	7 [2–38]	2 [1–5]	< 0.0001
Duration of hospitalization in ICU, median [IQR]	14 [9–25]	23 [12–56]	13 [9–22]	0.001
Death at Day 28, n (%)	10 (6)	2 (5)	8 (7)	1.00
Death at Day 90, n (%)	24 (15)	11(26)	13 (11)	0.04
Death at one year, n (%)	38 (24)	18 (43)	20 (18)	0.003

*BAD* bronchial anastomotic dehiscence, *BMI* Body Mass Index, *HBP* high blood pressure, *LT* lung transplantation, *COPD* chronic obstructive pulmonary disease, *PF* pulmonary fibrosis, *MV* mechanical ventilation, *SOFA* Sequential organ failure assessment, *SAPSII* Simplified acute physiological score II, *PGD* primary graft dysfunction

### Incidence and outcome of BAD

**Fig. 2:**
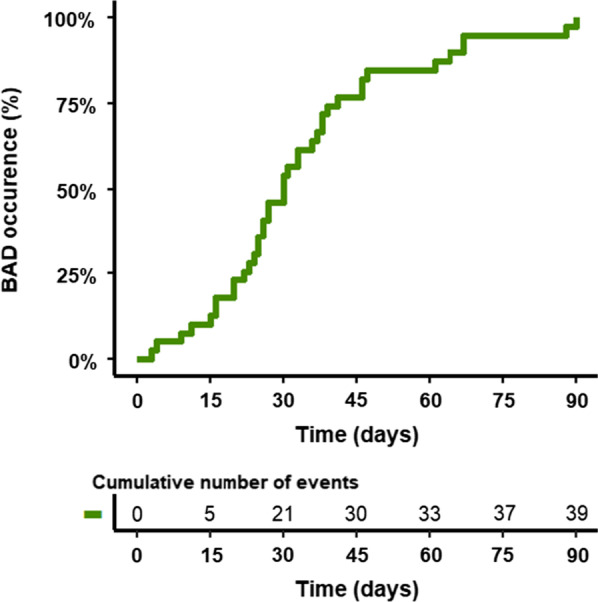
Delay for BAD occurrence during the first 90 days after LT

**Fig. 3:**
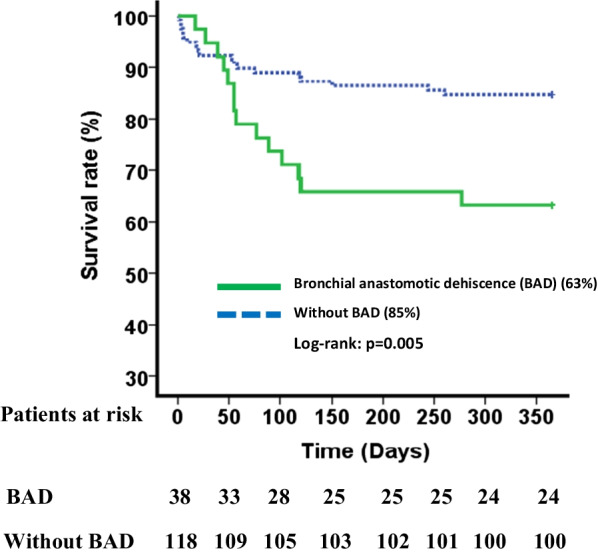
90-days survival of LT recipients according to BAD at Day 90

BAD occurred during the first 90 postoperative days in 42 (27%) patients. BAD occurred during the first month after surgery in 34/42 (81%) patients and after the first month in 8 (19%) patients. The delay of BAD occurrence is presented in Fig. 2. BAD was the main cause of death during the first 90 days after surgery in 7 (4%) patients and during the first year after LT in 15 (10%) patients. The 90-days survival of LT recipients depending on BAD is presented in Fig. 3. A total of 13 recipients (8% of the total cohort) died before Day 90 without having developed any BAD.

### ECMO support in the perioperative period

ECMO was used as a bridge to LT in 9 (6%) patients (veno-venous ECMO in 5 (56%) patients, veno-arterial in 4 (44%) patients). Veno-venous ECMO was converted into a veno-arterial device during the intraoperative period in 4 (80%) patients. ECMO support was used during and after surgery in 117 (75%) and 30 (26%) patients, respectively.

### Description of the perioperative haemodynamic status

During surgery, high-dosage catecholamine was administered in 50 (32%) patients. Vascular filling was > 30 mL per kilogram in 136 (87%) patients. Red blood cell transfusion of 5 units or more was administered in 32 (21%) patients. On ICU admission, hyperlactatemia was observed in 77 (49%) patients. Lactatemia at ICU admission was > 3 mmol/L in 37 (32%) patients.

### Implication of ECMO support and haemodynamic status in BAD occurrence in the first 90 days after LT

BAD occurrence depending on the perioperative haemodynamic status of the recipients is presented in Table 2. None of the data linked to the donor’s haemodynamic status were associated with BAD after LT. In univariate analysis, ECMO as a bridge to LT and during surgery were associated with BAD occurrence (*p* = 0.01 and 0.02, respectively). High-dosage catecholamine administration during surgery, catecholamine administration in the ICU and duration, postoperative ECMO support and duration, and hyperlactatemia at admission in the ICU were not associated with BAD occurrence. Sensitivity analysis after exclusion of the 13 patients who died before Day 90 from a cause other than BAD is presented as Additional file1: Table S1. Independent risk factors for BAD occurrence during the first 90 days after LT are presented in Table 3. ECMO as a bridge to LT was an independent risk factor for BAD (OR 4.84, 95% CI 1.05–22.4, *p* = 0.04). The characteristics of LT recipients with BAD depending on ECMO support as a bridge to LT and characteristics of LT recipients with ECMO as a bridge to LT depending on BAD occurrence are presented as Additional file1: Table S2A and S2B. Among the recipients who received ECMO as a bridge to LT, BAD occurred after a median duration of 7 days of ECMO support and after a median duration of 5 days of catecholamine administration.

**Table 2:** Haemodynamic status and BAD occurrence at Day 90, univariate analysis

	BAD at day 90	No BAD at day 90	p
	n = 42 (27)	n = 114 (73)	
*Haemodynamic status before LT*			
Catecholamine administration to the donor, n (%)	32 (84)	90 (86)	0.79
Catecholamine > 0.5 γ/kg/min to the donor, n (%)	6 (14)	14 (12)	0.79
Cardiac arrest of the donor, n (%)	13 (31)	33 (29)	0.84
RBC transfusion of the donor, n (%)	14 (37)	30 (29)	0.41
ECMO as a bridge to LT (recipient), n (%)	6 (14)	3 (3)	0.01
*Haemodynamic status during LT surgery*			
Catecholamine > 0.5 γ/kg/min during surgery, n (%)	13 (31)	37 (32)	1.00
ECMO support during surgery, n (%)	37 (88)	80 (70)	0.02
ECMO weaned in operating room, n (%)	21 (50)	56 (50)	1.00
Vascular filling > 30 mL/kg, n (%)	38 (91)	98 (88)	0.78
Vascular filling > 2500 mL, n (%)	38 (91)	100 (89)	1.00
RBC transfusion, n (%)	31 (74)	75 (66)	0.44
> 5 RBC units transfusion, n (%)	9 (22)	23 (20)	0.83
FFP transfusion, n (%)	30 (71)	70 (61)	0.27
Platelet transfusion, n (%)	15 (40)	29 (27)	0.15
Cardiac arrest during surgery, n (%)	1 (2)	6 (5)	0.68
*Haemodynamic status during hospitalization in ICU*			
Lactate on ICU admission > 2 mmol/l, n (%)	21 (50)	56 (49)	1.00
Lactate on ICU admission > 3 mmol/l, n (%)	9 (21)	29 (25)	0.68
Catecholamines administration during hospitalization in ICU, n (%)	38 (93)	107 (96)	0.39
Duration of catecholamine administration, days, median [IQR]	2 [1–3, 5]	1 [1–3]	0.16
Duration of ECMO support, days, median [IQR]	0 [0–3]	0 [0–1]	0.06
Atrial fibrillation, n (%)	18 (43)	28 (25)	0.05
AKI, n (%)	29 (69)	23 (20)	< 0.0001
KDIGO stage, median [IQR]	1 {0–2]	1 [0–2]	0.16
RRT, n (%)	5 (12)	11 (10)	0.77
Septic shock, n (%)	18 (43)	19 (17)	0.001
Haemorrhagic shock, n (%)	4 (10)	13 (11)	1
Cardiogenic shock, n (%)	5 (12)	7 (6)	0.31
MOF syndrome, n (%)	17 (42)	26 (23)	0.03
Cardiac arrest during hospitalization in ICU, n (%)	7 (17)	10 (9)	0.16

*BAD* bronchial anastomotic dehiscence, *LT* lung transplantation, *RBC* red blood cell, *ECMO* extracorporeal membrane oxygenation, *FFP* fresh frozen plasma, *ICU* intensive care unit, *IQR* interquartile range, acute kidney injury, *KDIGO* kidney disease improving global outcome, *RRT*, renal replacement therapy, *MOF* multiorgan organ failure

**Table 3:** Risk factors for BAD at Day 90, multivariate analysis

	OR	95%CI	p
ECMO as a bridge to LT	4.84	[1.05–22.4]	0.04
Intraoperative ECMO support	2.37	[0.82–6.88]	0.11
Bilateral LT	1.18	[0.48–2.94]	0.72
Acute cellular rejection	1.71	[0.74–3.97]	0.21
Septic shock	3.11	[1.33–7.29]	0.01

*BAD* bronchial anastomotic dehiscence, *OR* odds ratio, *CI* confidence interval, *ECMO* extracorporeal membrane oxygenation, *LT* lung transplantation

## Discussion

The first aim of this prospective study was to assess the association between perioperative ECMO support and BAD during the 90 days after LT in a 156 LT recipient monocentric cohort. BAD was observed in 42 (27%) patients during the first 90 days after LT and was the main cause of death in 22 (14%) patients. On multivariate analysis, ECMO as a bridge to LT and septic shock during hospitalization in the ICU were independent risk factors for BAD (*p* = 0.04 and 0.01, respectively).

The incidence of BAD was 27% in our cohort, representing one of the highest published rates. Indeed, prior studies established an incidence of anastomotic dehiscence between 1 and 10% [2, 7, 8]. We can hypothesize that the higher incidence in our cohort can be explained by the prospective nature of the study, allowing the recording of small, one-eyed, rapidly healing dehiscence, which could have been omitted in retrospective studies. The high frequency of bronchial endoscopic monitoring in our centre (every 48 h in the early postoperative period) could also explain this high incidence. Indeed, according to the ISHLT consensus statement on airway complications, 37% of cases of BAD are asymptomatic and accidentally discovered by bronchial endoscopy [2].

Another interesting characteristic of our cohort is the high mortality rate of LT recipients with BAD (airway complications were the main cause of death in 52% of patients with BAD). We can hypothesize that death occurred from multifactorial causes, including BAD (infectious complications, acute rejections, stage 3 PGD). Indeed, the mortality rate in our cohort was higher than worldwide published mortality rates (15% vs 6.2% at Day 90, 24% vs 13.6% at one year in a cohort of 20.294 transplants) [25]. This finding can be explained by the relatively high numbers of single LT and transplantation for PF, which are known risk factors for 1 year mortality [26].

Interestingly, the incidence of ECMO support during surgery in our cohort was higher than that in prior published studies (which reported ECMO support during surgery in 42–56% of the cases [19–21] vs 75% in our cohort). However, ECMO was weaned more frequently immediately after surgery in our cohort (74% vs 45% according to prior studies [21, 22], and the mean duration of ECMO support was also shorter (< 1 day in our cohort vs 1.5–3 days in published studies [21, 22].

In our study, multivariate analysis identified ECMO as a bridge to LT as an independent risk factor for BAD during the first 90 days after LT. To our knowledge, this association has never been described, but none of the published studies specifically assessed the impact of ECMO support on BAD occurrence. Interestingly, the effects of ECMO on regional circulations have been partially investigated, and neither ECMO-induced modifications in regional bronchial circulation nor bronchial mucosal oxygenation have ever been described. Several studies evaluating patients with septic and cardiogenic shocks demonstrated that despite macroscopic haemodynamic restauration, alterations in microcirculation can persist and are good predictor of mortality [27–30]. This has also been demonstrated in patients with refractory cardiogenic shock requiring venoarterial ECMO support [31]. We can assume that alterations in bronchial microcirculation in patients with ECMO as bridge to LT (so as recipients who present a septic shock during hospitalization in ICU), could favor the development of BAD—but this hypothesis has to be confirmed by further explorations.

A prior multicentric study retrospectively analysed anastomotic dehiscence in 18.122 LT recipients and found that the incidence of these complications remained stable at approximately 1.5% between 2008 and 2017, despite an increasing use of ECMO support [13]. This was not in favour of an impact of ECMO support on the occurrence of airway complications. However, the retrospective nature of this study may have underestimated the incidence of these complications (1.5% vs 27% in our cohort), resulting in an inability to detect differences. In light of our results, the adoption of enhanced bronchial monitoring protocols for patients with ECMO as a bridge to LT has to be discussed. Endoscopic methods for assessing local bronchial circulatory conditions and predicting anastomosis-related morbidity have already been described and have to be evaluated in LT [32]. In addition, shortening the duration of ECMO support could contribute to reducing the incidence of anastomotic complications.

In our study, the occurrence of septic shock in the ICU was associated with BAD. However, the observational design of the study cannot establish a cause–effect relationship. BAD can be both the consequence (imbalance between oxygen delivery and requirement, alteration of microcirculation) and the cause of septic shock (pleural infection).

In our study, none of the studied haemodynamic variables of the donor were associated with BAD during the first 90 days after LT. The impact of the haemodynamic status of the donor on BAD occurrence has never been studied. Our results suggest that the consequences of haemodynamic instability (administration of high-dosage catecholamine, cardiac arrest before organ donation) are not significant when bronchial circulation is not interrupted.

Our study presents several limitations. Firstly, the monocentric design of the study and the relatively short size of the cohort limit the extrapolation of the results, especially because of the small number of cystic fibrosis patients (1% vs 15% worldwide [33] and the high number of single LTs (33% vs 20% worldwide [33]. Secondly, the definition of haemodynamic instability (imbalance between oxygen supply and requirement) is quite vague. Indeed, a single variable (hypotension, changes in cardiac output, lactate measurement…) cannot characterize haemodynamic status of the recipient, because all of these variables are interrelated. That’s why we chose to study a large panel of clinical and biological variables (ECMO support and duration, catecholamine use and dosage, transfusion in order to characterize intraoperative bleeding, lactate on admission in ICU…) in order to characterize the haemodynamic status of the recipient. Thirdly, clinical practices (haemodynamic monitoring and optimization during surgery, bronchial endoscopic monitoring after LT, and management of bronchial complications) are variables in the different LT centres. Fourthly, our study assessed BAD, but the other airway complications (bronchial necrosis, stenosis, bronchomalacia) were not studied. Indeed, endoscopic constatations are operator-dependent, making it difficult to correctly collect these events. Fifthly, systemic haemodynamic status does not perfectly reflect local haemodynamic status at the anastomosis site. Finally, the observational nature of our study does not establish a cause-effect relationship between ECMO support as a bridge to LT and BAD. A higher severity of recipients with preoperative ECMO, resulting in more postoperative complications and higher mortality rates [34], can be the cause of BAD regardless of ECMO support.

## Conclusion

Bronchial anastomotic dehiscence remains a frequent life-threatening complication after LT. Risk factors for these complications are partially identified, especially the possible implication of perioperative haemodynamic instability. Our study identified ECMO as a bridge to LT and septic shock occurrence during hospitalization in the ICU as independent risk factors for BAD during the first 90 days after LT. These findings should encourage physicians to establish enhanced bronchial monitoring protocols for these high-risk patients. Considering the results of our study, ECMO support as bridge to LT should remain a salvage therapy. A pathophysiological support might help in the future to improve the outcome of LT recipients.

## Supplementary Information

**Additional file1**. **Table S1**: Haemodynamic status and BAD occurrence at Day 90, after exclusion of patients who died before Day 90 from a cause other than BAD, univariate analysis. **Table S2A**: Characteristics of LT recipients with BAD according to ECMO as a bridge to LT, univariate analysis. **Table S2B**: Characteristics of LT recipients with ECMO as a bridge to LT depending on BAD occurrence.

## Data Availability

Data are available on request from the corresponding author.

## Abbreviations

AKI: Acute kidney injury
BAD: Bronchial anastomotic dehiscence
BMI: Body mass index
COPD: Chronic obstructive pulmonary disease
ECMO: Extracorporeal membrane oxygenation
FFP: Fresh frozen plasma
ICU: Intensive care unit
ISHLT: International Society of Heart and Lung Transplantation
KDIGO: Kidney disease improving global outcome
LT: Lung transplantation
MOF: Multiorgan failure
MV: Mechanical ventilation
OR: Odds ratio
PAP: Pulmonary arterial pressure
PF: Pulmonary fibrosis
PGD: Primary graft dysfunction
RBC: Red blood cell
RRT: Renal replacement therapy
SOFA: Sepsis-related organ failure assessment
