# Supplementary material for: ECMO support as a bridge to lung transplantation is an independent risk factor for bronchial anastomotic dehiscence
Source: BMC Pulm Med. 2022 Dec 20;22:482. doi: 10.1186/s12890-022-02280-9 (PMC9764472; doi:10.1186/s12890-022-02280-9)
Supplement: Supplementary file 1 — Additional file1. Table S1: Haemodynamic status and BAD occurrence at Day 90, after exclusion of patients who died before Day 90 from a cause other than BAD, univariate analysis. Table S2A: Characteristics of LT recipients with BAD according to ECMO as a bridge to LT, univariate analysis. Table S2B: Characteristics of LT recipients with ECMO as a bridge to LT depending on BAD occurrence. [file 12890_2022_2280_MOESM1_ESM.docx]

**SUPPLEMENTAL DATA**

**TABLE S1:** Haemodynamic status and BAD occurrence at Day 90, after exclusion of patients who died before Day 90 from a cause other than BAD, univariate analysis

|  | BAD at Day 90 n=42 (27) | No BAD at Day 90 n=101 (73) | p |
| --- | --- | --- | --- |
|  |  |  |  |
| Haemodynamic status before LT |  |  |  |
| Catecholamine administration to the donor, n (%) | 32 (84) | 82 (86) | 0.79 |
| Catecholamine > 0.5 γ/kg/min to the donor, n (%) | 5 (12) | 23 (23) | 0.17 |
| Cardiac arrest of the donor, n (%) | 11 (26) | 32 (32) | 0.55 |
| RBC transfusion of the donor, n (%) | 14 (37) | 26 (27) | 0.30 |
| ECMO as a bridge to LT (recipient), n (%) | 6 (14) | 2 (2) | 0.008 |
| Haemodynamic status during LT surgery |  |  |  |
| Catecholamine > 0.5 γ/kg/min during surgery, n (%) | 11 (26) | 30(30) | 0.84 |
| ECMO support during surgery, n (%) | 37 (88) | 68 (67) | 0.01 |
| ECMO weaned in operating room, n (%) | 21 (50) | 51 (51) | 1.00 |
| Vascular filling > 30 ml/kg, n (%) | 38 (91) | 86 (87) | 0.78 |
| Vascular filling > 2500 ml, n (%) | 38 (91) | 87 (88) | 0.78 |
| RBC transfusion, n (%) | 31 (74) | 64 (64) | 0.25 |
| > 5 RBC units transfusion, n (%) | 9 (21) | 16 (16) | 0.47 |
| FFP transfusion, n (%) | 30 (71) | 59 (59) | 0.19 |
| Platelet transfusion, n (%) | 15 (40) | 22 (23) | 0.06 |
| Cardiac arrest during surgery, n (%) | 1 (2) | 3 (3) | 1.00 |
| Haemodynamic status during hospitalization in ICU |  |  |  |
| Lactate on ICU admission > 2 mmol/l, n (%) | 21 (50) | 47 (47) | 0.72 |
| Lactate on ICU admission > 3 mmol/l, n (%) | 9 (21) | 22 (22) | 1.00 |
| Catecholamine administration during hospitalization in ICU, n (%) | 38 (93) | 94 (96) | 0.42 |
| Duration of catecholamine administration, days, median [IQR] | 2 (1-4] | 1 [1-3] | 0.08 |
| Duration of ECMO support, days, median [IQR] | 0 [0-3] | 0 [0-0] | 0.01 |
| Atrial fibrillation, n (%) | 18 (43) | 25 (25) | 0.05 |
| AKI, n (%) | 25 (60) | 53 (52) | 0.47 |
| KDIGO stage, median [IQR] | 1 [0-2] | 1 [0-2] | 0.04 |
| RRT, n (%) | 5 (12) | 4 (4) | 0.12 |
| Septic shock, n (%) | 18 (43) | 12 (12) | <0.0001 |
| Haemorrhagic shock, n (%) | 4 (10) | 6 (6) | 0.48 |
| Cardiogenic shock, n (%) | 5 (12) | 5 (5) | 0.16 |
| MOF syndrome, n (%) | 17 (42) | 18 (18) | 0.01 |
| Cardiac arrest during hospitalization in ICU, n (%) | 7 (17) | 3 (3) | 0.01 |

BAD: bronchial anastomotic dehiscence; LT: lung transplantation; RBC: red blood cell; ECMO: extracorporeal membrane oxygenation; FFP: fresh frozen plasma; ICU: intensive care unit; IQR: interquartile range; acute kidney injury; KDIGO: kidney disease improving global outcome; RRT, renal replacement therapy; MOF: multiorgan organ failure.

**TABLE S2A:** Characteristics of LT recipients with BAD according to ECMO as a bridge to LT, univariate analysis

|  | **Overall**  **n=42** | **No ECMO as a bridge to LT**  **n=36 (86)** | **ECMO as a bridge to LT**  **n=6 (14)** | **p** |
| --- | --- | --- | --- | --- |
| Duration of ECMO support, days, median [IQR] | 0 [0, 3] | 0 [0, 3] | 2 [1, 3] | 0.13 |
| Delay of BAD occurrence, days, median [IQR] | 30 [22, 40] | 30 [24, 45] | 20 [16, 31] | 0.13 |
| Duration of catecholamine support before BAD, days, median [IQR] | 4 [2, 9] | 4 [2, 9] | 5 [2, 12] | 0.82 |
| Death at Day 90, n (%) | 11 (26) | 10 (28) | 1 (17) | 1 |
| Death at one year, n (%) | 18 (43) | 17 (47) | 1 (17) | 0.21 |

**TABLE S2B:** Characteristics of LT recipients with ECMO as a bridge to LT depending on BAD occurrence

|  | **Overall**  n = 9 | **No BAD**  n = 3 (33) | **BAD**  n = 6 (67) | **p** |
| --- | --- | --- | --- | --- |
| Duration of ECMO support before LT, days, med [IQR] | 5 [3, 8] | 5 [4, 7] | 5 [3, 8] | 0.90 |
| Death at Day 90, n (%) | 2 (22) | 1 (33) | 1 (17) | 1 |
| Death at one year, n (%) | 2 (22) | 1 (33) | 1 (17) | 1 |

LT: lung transplantation; ECMO: extracorporeal membrane oxygenation; BAD: bronchial anastomotic dehiscence
